# Overexpression of membrane-bound gluconate-2-dehydrogenase to enhance the production of 2-keto-d-gluconic acid by *Gluconobacter oxydans*

**DOI:** 10.1186/s12934-016-0521-8

**Published:** 2016-07-09

**Authors:** Kefei Li, Xinlei Mao, Liu Liu, Jinping Lin, Ming Sun, Dongzhi Wei, Shengli Yang

**Affiliations:** State Key Laboratory of Bioreactor Engineering, New World Biotechnology Institute, East China University of Science and Technology, Shanghai, 200237 China

**Keywords:** 2-keto-d-gluconic acid, *Gluconobacter oxydans*, Overexpression, Resting cell

## Abstract

**Background:**

2-keto-d-gluconic acid (2KGA) is widely used as a chemical intermediate in the cosmetic, pharmaceutical and environmental industries. Several microbial fermentation processes have been developed for production of 2KGA but these suffer from substrate/product inhibition, byproduct formation and low productivity. In previous work, we showed that 2KGA can be specifically produced from glucose (Glu) or gluconic acid (GA) by resting wild-type *Gluconobacter oxydans* DSM2003 cells, although substrate concentration was relatively low. In this study, we attempted to improve 2KGA productivity by *G. oxydans* DSM2003 by overexpressing the *ga2dh* gene, which encodes the membrane-bound gluconate-2-dehydrogenase enzyme (GA2DH).

**Results:**

The *ga2dh* gene was overexpressed in *G. oxydans* DSM2003 under the control of three promoters, P_*tufB*_, P_*ga2dh*_ or P_*ghp0169*_, respectively. Among the recombinant strains obtained, *G. oxydans_tufB_ga2dh* showed a similar growth rate to that of the control strain and displayed the highest specific productivity of 2KGA from GA, which was increased nearly twofold compared with that of the control strain during batch biotransformation. When biocatalysis conditions were optimized, with provision of sufficient oxygen during biotransformation, up to 480 g/L GA was completely utilized over 45 h by resting cells of *G. oxydans_tufB_ga2dh* and 453.3 g/L 2KGA was produced. A productivity of 10.07 g/L/h and a yield of 95.3 % were obtained. Overexpression of the *ga2dh* gene also significantly improved the conversion of Glu to 2KGA. Under optimized conditions, 270 g/L Glu was converted to 321 g/L 2KGA over 18 h, with a yield of 99.1 % and a productivity of 17.83 g/L/h. The glucose concentrations during the batch biotransformation and the 2KGA productivities achieved in this study were relatively high compared with the results of previous studies.

**Conclusions:**

This study developed an efficient bacterial strain (*G. oxydans_tufB_ga2dh)* for the production of 2KGA by overexpressing the *ga2dh* gene in *G. oxydans*. Supply of sufficient oxygen enhanced the positive effect of gene overexpression on 2KGA production. *Gluconobacter oxydans_tufB_ga2dh* is thus a competitive species for use in 2KGA production.

## Background

2-keto-d-gluconic acid (2KGA) is a noncorrosive mild organic acid, widely used in the cosmetic, pharmaceutical and environmental industries. It is of considerable interest that 2KGA is an intermediate for the generally regarded as safe (GRAS) antioxidant erythorbic acid (isoascorbic acid) and sodium erythorbate (EN) production [1].

2KGA can be produced from glucose by chemical process, enzymatic synthesis or microbial fermentation. During enzymatic or chemical synthesis, expensive enzymes or metal catalysts, such as Pt and Pb, are needed, and the production of by-products and low 2KGA yields is also disadvantageous [2]. To date, microbial fermentation appears to be the most efficient method for 2KGA production. *Gluconobacter, Pseudogluconobacter,Pseudomonas, Serratia and Klebsiella* spp. have been screened for 2KGA production [1, 3, 4]. The selectivity of glucose oxidation and the ratio of the acids produced vary widely among different strains and their culture conditions. The fermentation of various *Pseudomonas* species has been the most intensively studied for achieving good yields of 2KGA. The strain *P. fluorescens* AR4 was identified as having a higher efficiency for producing 2KGA than the previously obtained *S. marcescens*, *P. saccharoketogenes* or *P. aeruginosa* [5]. In semi-continuous culture, *P. fluorescens* AR4 could produce 444.96 g/L of 2KGA from a total of 476.88 g/L of glucose, resulting in a total productivity of 6.74 g/L/h and a yield of 0.93 g/g, which achieved the commercially acceptable levels of 2KGA. Nonetheless, the glucose tolerance of *P. fluorescens* AR4, as well as those of other reported 2KGA-producing strains, has been less than 200 g/L. Substrate inhibition remains a major concern in 2KGA fermentation when glucose or starch hydrolysates are used as carbon sources. The bioconversion of cassava-derived glucose to 2KGA was investigated using resting cells of immobilized *P. aeruginosa* [6]. Although the immobilized bacteria were successfully reused over a period of 2 weeks for the continuous production of 2KGA, with a good molar yield of 94 %, the final 2KGA titer was 35 g/L and the production rate was 0.55 g/L/h, which were relatively low.

The genus *Gluconobacter* are well known for their ability to rapidly but incompletely oxidize various sugars and sugar alcohols, and have therefore been used in several industrial oxidative fermentation processes [7–9]. Most *Gluconobacter* species can produce 2KGA from d-glucose via d-gluconic acid (GA) by two membrane-bound enzymes, glucose dehydrogenase (GDH) and gluconate-2-dehydrogenase (GA2DH). These enzymes are linked to the respiratory chain located in the cytoplasmic membrane facing the periplasm [10, 11], and accumulate the corresponding oxidized products almost completely in the culture medium. *Gluconobacter* species therefore have been exploited to establish the production process for 2KGA. However, byproducts 5KGA and/or 2,5-diketo-d–gluconate are also observed during the oxidation of glucose or GA in most *Gluconobacter* species [11, 12]. This not only lowers the final 2KGA productivity but also hinders downstream separation and purification of the product, and increases the production cost. The product formation pattern upon glucose oxidation depends upon the individual strains used. Both Weenk et al. [13] and Herrmann et al. [14] found that different *G. oxydans* strains differ in the ratio of their products from glucose. In addition, the medium and cultivation conditions influence the individual product yields. Weenk et al. also found that the influence of pH on product formation was significant [13]. For industrial production, it is preferable to engineer *G. oxydans* to enhance their catalytic properties during biotransformation. Although some early attempts were made to increase the production of GA or exclusively 5KGA [15, 16], little progress has been achieved in 2KGA accumulation by Gluconobacter strains. Therefore, recombinant strains suitable for 2KGA production on an industrial scale remain to be investigated.

In our previous study, we obtained one strain (*G. oxydans DSM2003*) with a high conversion rate and excellent selectivity towards d-glucose oxidation. We noticed that resting and growing cells of *G. oxydans* DSM2003 differ in their ratios of 2KGA and 5KGA formation. During microbial fermentation, most d-glucose or GA as a carbon source was transformed to 2KGA by growing cells, accompanied by nearly the same amounts of 5KGA (50 % of the total keto-d-gluconate). However, with resting cells of *G. oxydans* DSM2003, d-glucose or GA were exclusively converted to 2KGA. During batch bioconversion, 1365 mmol/L (300 g/L)GA was converted to 2KGA with a high yield of >97 % and a productivity of 74 mmol/L/h (16.73 g/L) by 80 g/L resting cells (wet weight) [17]. Therefore, the strain *G. oxydans* DSM2003 was chosen for 2KGA production. Bioconversion of GA to 2KGA using resting cells was used because of its selective, high-yield production and easy product extraction. In this work, the *ga2dh* gene, which encodes GA2DH, was overexpressed in *G. oxydans DSM2003* with different promoters to enhance the production of 2KGA. The optimization of 2KGA production through catalysis by resting cells of the best recombinant strain, *G. oxydans_tufB_ga2dh*, was investigated.

## Results

### Overexpression of ga2dh in *G. oxydans DSM2003*

Efficient expression of *ga2dh* (gox1230-1232) was essential for enhancing the 2KGA production in *G. oxydans DSM2003*. A well-characterized promoter is a prerequisite for the overexpression of an enzyme. To achieve the optimum recombinant strains, three different promoters (*G. oxydans_tufB*, *G. oxydans_ gHp0169* and the native promoter of the *ga2dh* gene) were introduced into the broad-host-range vector pBBR1MCS5 for expression of the *ga2dh* gene. *G. oxydans_tufB* and *G. oxydans_ gHp0169* were confirmed to be strong promoters for cloning and expression of homologous and heterologous genes in *G. oxydans* [18]. The three recombinant strains (*G. oxydans_tufB_ga2dh G. oxydans_g2adh_ga2dh,* and *G. oxydans_gHp0169_ga2dh*) and the control strain *G. oxydans_*pBBR1MCS5 were cultured in shaking flasks, and the activities of the obtained resting cells toward GA for 2KGA production were compared. Growth behaviors of the recombinant strains were similar to that of *G. oxydans_*pBBR1MCS5, but the biomass at the late-log phase was slightly lower than that of the control strain (Table 1). During the biocatalysis of GA by resting cells, all of the *ga2dh*-overexpressing strains produced concentrations of 2KGA higher than that of the control strain. Amongst these strains, *G. oxydans_tufB_ga2dh* and *G. oxydans_gHp0169_ga2dh* exhibited the highest specific productivities of 2KGA (0.83 and 0.85 g/g/h), about 100 % higher than that of *G. oxydans_*pBBR1MCS5 (0.42 g/g/h).

**Table 1:** Cell growth of different *G. oxydans* strains and their specific2KGA productivities

	Biomass (g/L)	Specific productivity (g/g_(wet wt)_/h)
*G. oxydans_*pBBR1MCS5	29 ± 2	0.42
*G. oxydans_tufB_ga2dh*	29 ± 3	0.83
*G. oxydans_g2adh_ga2dh*	28 ± 2.5	0.61
*G. oxydans_gHp0169_ga2dh*	25 ± 2.5	0.85

**Fig. 1:**
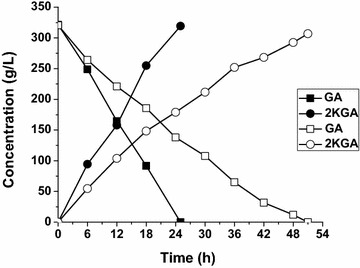
Comparison of 2KGA production by *G. oxydans_*pBBR1MCS5 and *G.oxydans_tufB_ga2dh.* The biotransformations were carried out in 7-L fermenterat 30 °C, pH 5.8, 600 rpm, aeration rate 8 L/min and cell concentration 30 g_(wet wt)_/L. *Gluconobacter oxydans_*pBBR1MCS5 (*blank*), *G. oxydans_tufB_ga2dh* (*filled*)

Concerning the biomass and specific productivity of 2KGA production, the optimal strain *G. oxydans_tufB_ga2dh* was selected and batch bioconversion of GA by resting cells was conducted in a 7-L fermenter with a GA concentration increased to 320 g/L. During the bioconversion, the agitation speed and aeration rate were controlled at 600 rpm and 8 L/min, respectively. pH was controlled at 5.8 using 4 mol/L NaOH solution. As shown in Fig. 1, almost all the GA was converted to 2KGA by 30 g_(wet wt)_/L cells of the engineered strain in about 25 h, generating 319 g/L 2KGA at a productivity of 12.76 g/L/h. In contrast, the control strain *G. oxydans_*pBBR1MCS5 required 51 h to complete this reaction,generating 307 g/L 2KGA at a productivity of 6.02 g/L/h. The results show that enhanced expression of the *ga2dh* gene in *G. oxydans* under the control of *tufB* promoter efficiently improved the production yield of 2KGA from GA.

**Fig. 2:**
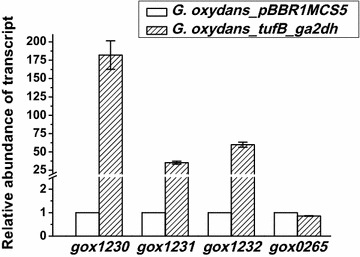
Relative transcriptional abundance of the *ga2dh* and *gdh* gene *G. oxydans_*pBBR1MCS5 (*blank*) and *G. oxydans_tufB_ga2dh* (*shadow*)

As expected, the transcriptional levels of the *ga2dh* gene in the engineered strain (*G. oxydans_tufB_ga2dh*) were significantly enhanced (Fig. 2). The *ga2dh* expression level obtained was normalized in the control strain *G. oxydans*_pBBR1MCS5. The transcriptional abundance of the three subunits (*gox*1230, 1231 and 1232) of the *ga2dh* gene in *G. oxydans_tufB_ga2dh* were 180, 35 and 60-fold higher than those of the control strain *G. oxydans*_pBBR1MCS5 respectively. But the transcriptional abundance of the *gdh* gene (*gox*0265) in *G. oxydans_tufB_ga2dh* was about 85 % of that in *G. oxydans*_pBBR1MCS5.

### Optimization of the biocatalysis conditions by resting *G. oxydans_tufB_ga2dh* cells

To explore the potential of *G. oxydans_tufB_ga2dh* in 2KGA production and achieve a high production titer, the biocatalysis conditions for 2KGA production from GA were optimized. In a 7-L fermenter, pre-experiments had proven that the optimum reaction temperature and pH were 30 °C and 5.8, respectively.

**Fig. 3:**
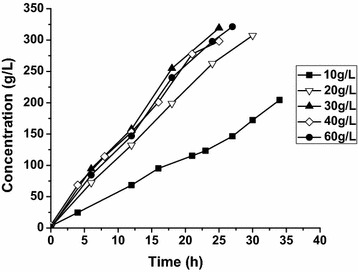
Effect of cell concentration on 2KGA production. The biotransformations were conducted by 10, 20, 30, 40 and 60 g/L *G. oxydans_tufB_ga2dh* resting cells, respectively

A suitable amount of cell content is necessary for high 2KGA production and the economic feasibility of the bioprocess. To determine the effect of the cell content on 2KGA production, various concentrations of resting *G. oxydans_tufB_ga2dh* cells were used to catalyze 320 g/L GA. As shown in Fig. 3, 2KGA production and the reaction rate increased with the cell concentration increasing to 30 g_(wet wt)_/L, and then were nearly constant as cell concentration continued to increase. In the presence of 30 g/L resting cells, 2KGA accumulation linearly increased and reached a maximum after 25 h, at which time all GA had been converted to 2KGA, resulting in the highest productivity of 12.76 g/L/h.

**Fig. 4:**
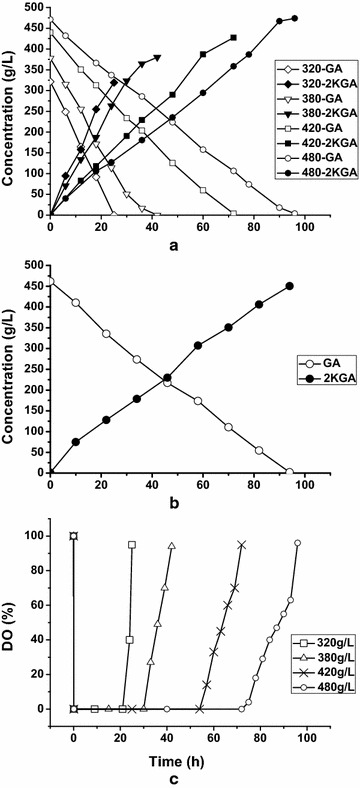
Effect of initial GA concentration on 2KGA production. **a** The initial GA concentration were 320, 380, 420 and 480 g/L, respectively. Cell concentration was 30 g_(wet wt)_/L. GA (*open*); 2KGA (*filled*), **b** Initial GA concentration 480 g/L; cell concentration 60 g_(wet wt)_/L. GA (*open*); 2KGA (*filled*). **c** DO profile during the batch bioconversion, cell concentration 30 g_(wet wt)_/L

The effect of initial GA concentration on 2KGA production was also investigated. Reactions with four different GA concentrations (320, 380, 440 and 480 g/L) were conducted with 30 g_(wet wt)_/L resting cells at pH 5.8 and 30 °C. Almost all the GA at different concentrations were converted to 2KGA with yields close to 100 %, but the productivity decreased with increasing GA concentration, because of the extension of reaction time when the substrate concentration was increased (Fig. 4a). The 2KGA productivities were 12.76, 9.04, 5.93 and 4.93 g/L/h, at initial GA concentrations of 320, 380, 440 and 480 g/L, respectively. At a high initial GA concentration of 480 g/L, an enhanced concentration of resting cells (60 g_(wet wt)_/L) had no effect on the productivity for 2KGA production (Fig. 4b). Both GA conversion rate and 2KGA production rate with 60 g_(wet wt)_/L resting cells were identical with those at 30 g_(wet wt)_/L resting cells.

Given that the formation of 2KGA from GA requires oxygen as the final acceptor of electrons formed during the oxidation of GA, oxygen conditions were maintained via a high agitation speed (600 rpm) and air flow rate (8 L/min) throughout the batch bioconversion period. However, as shown in Fig. 4c, it was observed that when the reaction started, dissolved oxygen (DO) sharply decreased to 0 % air saturation, and then remained constant (less than 0 %) until the latter stages of the reaction (about 20 % of GA remaining). This may imply that DO is an important factor influencing the conversion of GA to 2KGA.

**Fig. 5:**
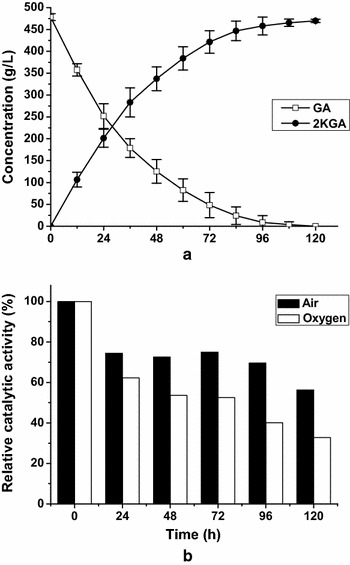
Effect of DO control strategy on bioconversion of GA to 2KGA. **a** Time course of GA consumption and 2KGA production with oxygen supply **b** Relative activities of resting cells during bioconversion

The main factors controlling DO concentration during biotransformation are the degree of agitation, gas flow rate and oxygen partial pressure in the supplied gas. Because of the limitations of the fermenter design, the agitation speed and gas flow rate could not be increased further. Thus, to increase DO levels, oxygen instead of air was supplied continuously to support the oxidation of 480 g/L GA by 30 g_(wet wt)_/L resting cells. Under this condition, the agitation speed and oxygen flow rate were controlled at 600 rpm and 1 L/min, respectively. The DO level in the reaction mixture was maintained above 100 % throughout the process. The time course for GA consumption and 2KGA production are shown in Fig. 5a. During the first 24 h of batch bioconversion, approximately 50 % of GA was linearly reduced and produced 199.42 ± 20.34 g/L 2KGA. Both 2KGA production and the conversion rate of GA to 2KGA were higher than those in bioconversion experiments under continuous air supply, in which a 2KGA titer of 129.42 ± 3.43 g/L and conversion rate of 27.4 % were obtained at 24 h (Fig. 4a). After 24 h, a continuous rise in 2KGA titer was accompanied by a gradual decrease in GA levels, as observed by declines in the product formation and substrate consumption rates. By 108 h, all GA was completely transformed into 2KGA at a level of 461.09 g/L with a productivity of 4.27 g/L/h. To determine the reasons for this behavior, samples of resting cells in the reaction mixture were taken at different reaction times and the relative activities toward GA were determined. The catalytic activities of the samples at the beginning of the reactions from air supply experiments or oxygen supply experiments were set at 100 %. As shown in Fig. 5b, the catalytic activities of the resting cells in these two experiments decreased with the extension of reaction time; however, there was a more marked loss of activity in the oxygen supply experiment than in the air supply experiment. Catalytic activities of resting cells in the oxygen supply experiment decreased by 40 % in 24 h, and showed 33 % activity at the end of bioconversion. The results clearly reveal that high oxygen levels suppressed the oxidative activity of resting cells toward GA, and an excess of oxygen during bioconversion may result in decreasing productivity. Therefore, an optimal oxygen level is important for high 2KGA productivity.

**Fig. 6:**
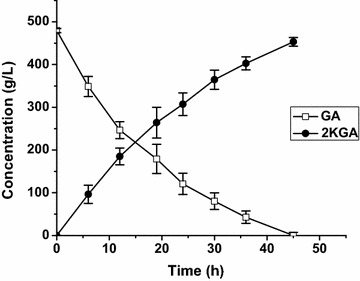
Bioconversion of GA to 2KGA with oxygen supply and enhanced cell content (60 g_(wet wt)_/L)

In the case of enhanced oxygen levels via continuous supply of oxygen (Fig. 6), cell content in the reaction mixture was also increased to 60 g_(wet wt)_/L to make up for the loss of activities of cells. As expected, the conversion time was considerably shortened. By 45 h, 480 g/L GA was completely exhausted, and the 2KGA titer reached about 453.3 g/L, generating a productivity of 10.07 g/L/h, which is 135.8 % higher than that achieved using 30 g/L resting cells.

### Bioconversion of glucose to 2KGA by *G. oxydans_tufB_ga2dh*

**Fig. 7:**
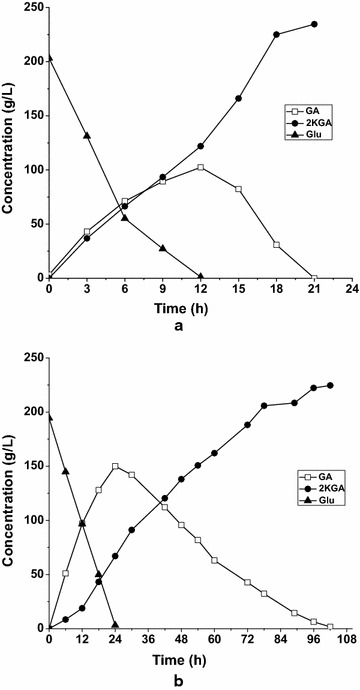
Comparison of 2KGA production from glucose. The biotransformations were carried out in 7-L fermenter at 30 °C, pH 5.8, 600 rpm, aeration rate 8 L/min, initial Glu concentration 200 g/L and cell concentration 60 g_(wet wt)_/L. **a** *G. oxydans_tufB_ga2dh* **b** *G. oxydans*_ pBBR1MCS5

Overexpression of the *ga2dh* gene in *G. oxydans* could significantly improve the productivity and 2KGA production from GA, and enhance the product formation rate from glucose. As shown in Fig. 7, the profiles for 2KGA production from glucose by *G. oxydans_tufB_ga2dh* and the control strain *G. oxydans*_pBBR1MCS5 were similar, but the glucose conversion rates and 2KGA formation rates were evidently different. As shown in Fig. 7a, 200 g/L glucose was consumed rapidly by 60 g/L resting *G. oxydans_tufB_ga2dh* cells, which was accompanied by an increase in 2KGA and GA accumulation. After all glucose was fully depleted at 12 h, GA accumulation reached a maximum of 102.42 g/L, with a glucose conversion rate of 16.67 g/L/h. However, using the same amount of resting cells of the control strain *G. oxydans_*pBBR1MCS5, the glucose conversion time was extended to 24 h, corresponding to a lower glucose conversion rate of 7.96 g/L/h (Fig. 7b). During the second period of GA conversion to 2KGA, all GA produced was further converted to 2KGA by *G. oxydans_tufB_ga2dh* within 12–21 h (i.e. elapsed 9 h) with a GA conversion rate of 11.38 g/L/h, which was about fivefold higher than that obtained using *G. oxydans*_pBBR1MCS5. 150.0 g/L GA produced was gradually decreased by *G. oxydans*_pBBR1MCS5 cells and was fully consumed over a long period of time (24–102 h, elapsed 78 h), resulting in a low GA conversion rate of 1.92 g/L/h. It was also demonstrated that overexpression of the *ga2dh* gene improves the conversion of GA to 2KGA. Unexpectedly, overexpression of the *ga2dh* gene significantly enhanced the conversion of glucose to GA. Overall, the final 2KGA titer reached 234.6 g/L at 21 h during the batch bioconversion of glucose by the engineered strain, corresponding to a productivity of 11.17 g/L/h, which amounted to a 407 % increase compared with that obtained using the control strain *G. oxydans*_pBBR1MCS5.

**Fig. 8:**
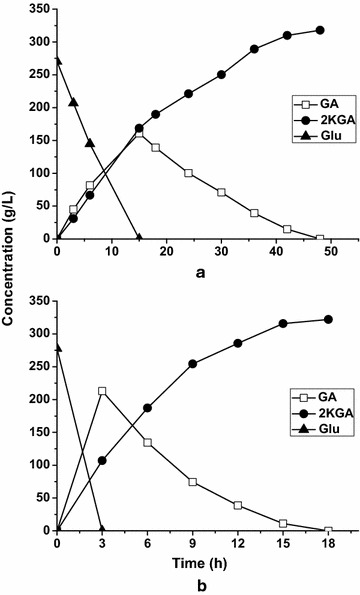
Comparison of 2KGA production from glucose by *G. oxydans_tufB_ga2dh.* Initial Glu concentration 270 g/L; Cell concentration 60 g_(wet wt)_/L. **a** Air **b** O_2_

When the glucose concentration was increased to 270 g/L, full conversion of glucose was observed at 15 h by 60 g/L resting cells of *G. oxydans_tufB_ga2dh*, and the 2KGA titer reached a maximum of 318 g/L at 48 h, giving a productivity of 6.63 g/L/h (Fig. 8a). During the overall reaction process, we also found that DO levels in the fermenter remained below 0 %. Because sufficient oxygen supply could enhance 2KGA productivity during GA conversion, oxygen instead of air was supplied continuously to support the oxidation of 270 g/L glucose at the same cell mass (Fig. 8b). As expected, the reaction time under oxygen supply was significantly decreased compared with that when air was used as the electron acceptor. The glucose conversion rate during the first period of GA formation and the GA conversion rate in the second period of GA conversion to 2KGA were increased by 400 and 268.8 %, respectively. All of the supplied glucose was converted to 321 g/L 2KGA over 18 h by the constructed strain *G. oxydans_tufB_ga2dh*, corresponding to a productivity of 17.83 g/L/h. Both the glucose concentration during batch biotransformation and the 2KGA productivity in this study were relatively high compared with those achieved by *Pseudomonas fluorescens* [5, 19, 20].

## Conclusions

Currently, almost all 2KGA is obtained by microbial fermentation; however, there are a number of problems with this process, including by-product (i.e., gluconate and 5KGA) formation, substrate/product inhibition, and modest productivity resulting from the long fermentation times required. A more economical process for 2KGA production has been a long sought after goal. In the present study, bioconversion of GA or glucose using resting cells of *G. oxydans* DSM2003 was developed to produce the sole product 2KGA. After overexpression of the *ga2dh* gene in the *G. oxydans* DSM2003 strain, the 2KGA productivities from GA (Fig. 1), glucose (Fig. 7) were enhanced by 112.5 and 407 %, respectively. DO concentration during the bioconversion of GA was identified as a key factor in 2KGA production by the engineered strain *G. oxydans_tufB_ga2dh*. When sufficient but not excessive oxygen was supplied during batch bioconversion, 453.3 g/L 2KGA was produced from 480 g/L GA with a yield of 95 % and a productivity of 10.07 g/L/h and 321 g/L 2KGA was produced from 270 g/L glucose with a yield of 99 % and a productivity of 17.83 g/L/h. The proposed efficient bioconversion process for 2KGA production by *G. oxydans_tufB_ga2dh* is potentially competitive in terms of 2KGA titer, yield and productivity, compared with reported microbial fermentation or bioconversion processes.

## Methods

### Materials

All chemicals and reagents were obtained from commercial sources. Oligonucleotides were synthesized by Generay (Shanghai, China). Restriction endonucleases, Taq DNA polymerase, and PCR reagents were purchased from Fermentas (Shanghai, China). DNA ligation kit was purchased from Takara (Dalian, China). GA (sodium salt) was purchased from Xiwang (Binzhou, China). Glucose, KH_2_PO_4_, MgSO_4_·7H_2_O, H_3_PO_4_, was purchased from Sinopharm (Shanghai, China). Sorbitol was purchased from Roquette (Lianyungang, China). Yeast extract was purchased from Oxoid (Hampshire, UK).

### Microorganisms and culture conditions

Strains used in this study are listed in Table 2. *Escherichia coli DH5α* was cultivated in lysogeny broth (LB) medium or on LB agar plates at 37 °C. *Gluconobacter oxydans* was grown in a medium containing 80 g/L sorbitol, 20 g/L yeast extract, 1 g/L KH_2_PO_4_, 0.5 g/L MgSO_4_·7H_2_O, 0.1 g/L glutamine and 25 μg/mL cefoxitin at 30 °C in 1-L Erlenmeyer fl asks (200 mL culture volume) on an incubator shaker (Hualida, Taichang, China) with a stirring rate of 200 rpm or in a 7-L Fermenter (4 L culture volume) with pH and aeration control. The pH was adjusted to pH 5.5 with H_3_PO_4_ before sterilization. Addition of 25 μg/mL Gentamicin was used for plasmid maintenance in *G. oxydans* and *E. coli.*

**Table 2:** Plasmid, strain or primer

plasmid, strain or primer	Description or primer sequence	Source or added site^a^
Plasmid		
pRK2013	Km^R^, mob^+^, tra^+^, helper plasmid	Laboratory stored
pBBR1MCS5	Broad-host-range cloning vector; *mod, rep, Gm* ^*R*^ *, lacZ*	[21]
pBBR1MCS5-P_*tufB*_-*ga2dh*	pBBRMCS5 derivative expressing *gox1230*-*gox1232* containing a *tufB* promoter, *Gm* ^*R*^	This study
pBBR1MCS5-P_*ga2dh*_-*ga2dh*	pBBRMCS5 derivative expressing *gox1230*-*gox1232* containing a *ga2dh* promoter, *Gm* ^*R*^	This study
pBBR1MCS5-P_*gHp0169*_-*ga2dh*	pBBRMCS5 derivative expressing gox1230-gox1232 containing a *gHp0169* promoter, *Gm* ^*R*^	This study
Strain		
*E. coli* DH5α	F-, ϕ80, lacZ∆M15, ∆(lacZYA-argF), U169, endA1, recA1, hsdR17(r_k_-m_K_+), supE44, λ^−^, thi-1, gyrA96, relA1, phoA	Tiangen, Beijing, China
*G. oxydans*	Wild type, cef^R^	DSM2003
*G. oxydans*_pBBR1MCS5	*G. oxydans* contain plasmid pBBR1MCS5	This study
*G. oxydans_tufB_ga2dh*	*G. oxydans* contain plasmid pBBR1MCS5-P_*tufB*_-*ga2dh*, Cef^R^, Gm^R^	This study
*G. oxydans_g2adh_ga2dh*	*G. oxydans* contain plasmid pBBR1MCS5-P_*ga2dh*_-*ga2dh,* Cef^R^, Gm^R^	This study
*G. oxydans_gHp0169_ga2dh*	*G. oxydans* contain plasmid pBBR1MCS5-P_*gHp0169*_-*ga2dh,* Cef^R^, Gm^R^	This study
Primer		
*ga2dh*f	CTATCTAGAGGAGAAACCTGTGCCCCCCATG	*Xba*I
*ga2dh*r	GAGGGATCCTTCAGTTCAGTGAGACCGCATCATC	*Bam*HI
P_*tufB*__f	ACTGAGCTCCGATGGTAAGAAATCCACTGC	*Sac*I
P_*tufB*__r	ATATCTAGACCAAAACCCCGCTCCACC	*Xba*I
P-*ga2dh*_f	CACTCTAGACAGAACCAGTGGCCGCCCCGACAAC	*Xba*I
P-*ga2dh*_r	GAGCTGCAGTTCAGTTCAGTGAGACCGCATCATC	*Pst*I
P-*gHp0169*_f	ATAGAGCTCTGAAAGCGGCTGGCGCGT	*Sac*I
P-*gHp0169*_r	GCATCTAGAGCGGAAGGCGTTATACCCTGA	*Xba*I
rtPCR primers		
16s-RNA_f	GCGGTTGTTACAGTCAGATG	
16s-RNA_r	GCCTCAGCGTCAGTATCG	
gox1230_f	GCTGGAGCGAGGAAGACATC	
gox1230_r	CGGGTGGCAGACTTTTGAG	
gox1231_f	CCAGAACCTGTCCCAGTCCAC	
gox1231_r	CGACAACATTACGGCAAAGG	
gox1232_f	CGACAACATTACGGCAAAGG	
gox1232_r	GCCAGGGAAACCCAGCAT	
gox0265_f	AATGTTCGAGTGGGGTGGTC	
gox0265_r	CCCTTGGCGTCCTTTTCAG	

^a^Restriction endonuclease sites underlined

All the sorbitol mediums mentioned above were sterilized by autoclaving at 121 °C for 20 min. Preculture of bacterium was inoculated with a single cell colony from an agar plate medium and cultured in a 500-mL orbital shaker containing 100 mL liquid medium (200 rpm, 30 °C) until the late exponential growth phase (20 h). The seed culture was repeated twice to adapt the culture to the fermentation environment. An inoculum concentration of 10 % (v/v) was added to the fermenter and grown at 30 °C for 20 h.

### Construction of recombinant plasmids

The *tufB* and *gHp0169* promoters were designed as described previously [21]. The *ga2dh* promoter from *G. oxydans* DSM2003 was sequenced and found to have the same sequence as that from *G. oxydans* 621H [22].The *ga2dh* promoter sequence could be found in Genebank (Genbank, CP000009.1, 1343338-1343689).

Restriction enzyme digestion, DNA ligation, and other DNA modifications were performed according to the manufacturer’s recommendations. Genomic DNA from *G. oxydans* DSM2003 was isolated by TIANamp Bacteria DNA Kit (TIANGEN) and used as a template for the amplification of *gox1230*-*gox1232* (Genbank, CP000009.1, *gox1230*, 1339527-1340840; *gox1231*, 1340837-1342615; *gox1232*, 1342617-1343327), *tufB* promoter and *gHp0169* promoter, with primers listed in Table 1. The resulting *tufB* promoter/*gHp0169* promoter and plasmid pBBR1MCS5 were doubly digested with restriction endonucleases *Sac*I and *Xba*I and ligated to construct the plasmids PBBR1MCS5-P_*tufB*_/P _*gHp0169*_. The constructed plasmids and the 3812 bp amplicon of *ga2dh* were doubly digested with restriction endonucleases *Xba*I and *Bam*HI and ligated to construct the plasmids pBBR1MCS5-P_*tufB*_-*ga2dh* and pBBR1MCS5-P _*gHp0169*_-*ga2dh.*

Moreover, a 4164 bp fragment encoding the *ga2dh* gene with its own promoter were PCR-amplified from genomic DNA of *G. oxydans* DSM2003 with the primers P-*ga2dh*_f/P-*ga2dh*_r. The resulting amplicon *and* PBBR1MCS5 were then doubly digested with restriction endonucleases *Xba*I and *Pst*I and ligated to construct the plasmids pBBR1MCS5-P_*ga2dh*_-*ga2dh*.

Constructs were transformed into *E. coli* DH5α. Positive transformants were sequenced by the Majorbio (Shanghai, China). The correct inserts were transformed into *G. oxydans* DSM2003 cells with the help of *E. coli* containing plasmid pRK2013. Transformation of *G. oxydans* was performed by triparental mating [23]. The positive transformants of *G. oxydans* were selected on agar media (as mentioned above) containing cefoxitin and gentamicin to final concentrations of 50 and 25 μg/mL, respectively. As a control, empty vector PBBR1MCS5 was also transformed into *G. oxydans* DSM2003 cells.

### Detection of gene expression by quantitative real-time PCR(qRT-PCR)

To measure the gene expression level of *ga2dh* and *gdh* (Genbank, CP000009.1, 280284-282710), real-time reverse transcription PCR (RT-PCR) was carried out. An inoculum concentration of 1 % (v/v) of the *G. oxydans* strains was added to 50 mL sorbitol medium in 250-mL shake flasks until the late exponential phase (16 h) at 30 °C and 200 rpm on a rotary shaker. After that, the cells were harvested by centrifugation at 10,000×*g* and 4 °C for 10 min for total RNA isolation. Total RNA was extracted by RNAiso reagent (Takara) and then treated with DNase I (Takara). The reverse transcription was carried out to obtain the cDNA, using total RNA as the template, with a first-strand cDNA synthesis kit (Promega, Madison, WI, USA). Quantitative gene expression analysis was performed by quantitative real-time PCR, which was carried out with the StepOnePlus™ Real-Time PCR System (Applied Biosystems, Foster City, CA, USA) using primers as listed in Table 1. The 16S rRNA gene was used as an internal standard.

### Whole-cell biotransformation of GA to 2KGA

Different strains of *G. oxydans* cells cultivated in sorbitol media in the 7-L fermenter (pH 5.5, 30 °C) were harvested by centrifugation (10,000×*g*, 20 min, 4 °C), and then washed with phosphate buffer (pH 6.0). Bioconversions were carried out in 100-mL shake flasks (10 mL transformation volume) which contained 10 g/L resting cells and 40 g/L GA, or in the 7-L fermenter (1.5 L transformation volume) containing resting cells and GA/Glu at 30 °C, pH 5.8. Samples were taken at suitable intervals, and the supernatants were obtained by centrifuging at 10,000×*g* and 4 °C for 10 min.

### Determination of substrate and product concentrations

GA (gluconate) and 2KGA (2-keto-d-gluconate) were determined by high-performance liquid chromatography (HPLC). The substances were separated with an IC Sep ICE-Coregel 87H3 reversed-phase HPLC column (Transgenomic, New Haven, CT, America) at a flow rate of 0.4 mL/min and 0.008 N H_2_SO_4_ was used as an eluant at 35 °C. GA and 2KGA were detected at 210 nm [24], and the concentrations were determined by comparison with corresponding calibration lines (1–100 mM for gluconate, 1–100 mM for 2-keto-d-gluconate).The peak maxima were: GA 15.9 min, 2KGA, 13.8 min.

## Abbreviations

2KGA: 2-keto-d-gluconic acid
GA: gluconic acid
Glu: glucose
*ga2dh*: membrane-bound gluconate-2-dehydrogenase gene
GA2DH: membrane-bound gluconate-2-dehydrogenase enzyme
*gdh*: membrane-bound glucose-dehydrogenase gene
DO: dissolved oxygen
